# Elevated SARS-CoV-2-Specific Antibody Levels in Patients with Post-COVID Syndrome

**DOI:** 10.3390/v15030701

**Published:** 2023-03-08

**Authors:** Christopher Hackenbruch, Yacine Maringer, Christian M. Tegeler, Juliane S. Walz, Annika Nelde, Jonas S. Heitmann

**Affiliations:** 1 Clinical Collaboration Unit Translational Immunology, German Cancer Consortium (DKTK), Department of Internal Medicine, University Hospital Tübingen, Otfried-Müller-Str. 10, 72076 Tübingen, Germany; 2 Cluster of Excellence iFIT (EXC2180) “Image-Guided and Functionally Instructed Tumor Therapies”, University of Tübingen, Röntgenweg 11, 72076 Tübingen, Germany; 3 Department for Peptide-Based Immunotherapy, University and University Hospital Tübingen, Otfried-Müller-Str. 10, 72076 Tübingen, Germany; 4 Institute for Cell Biology, Department of Immunology, University of Tübingen, Auf der Morgenstelle 15, 72076 Tübingen, Germany; 5 Department of Obstetrics and Gynecology, University Hospital of Tübingen, Calwerstraße 7, 72076 Tübingen, Germany

**Keywords:** SARS-CoV-2, COVID-19, post-COVID syndrome, humoral immune response

## Abstract

With the routine use of effective severe acute respiratory syndrome coronavirus 2 (SARS-CoV-2) vaccines, the number of life-threatening coronavirus disease 2019 (COVID-19) courses have largely been reduced. However, multiple COVID-19 convalescents, even after asymptomatic to moderate disease, suffer from post-COVID syndrome, with relevant limitations in daily life. The pathophysiologic mechanisms of post-COVID syndrome are still elusive, with dysregulation of the immune system suggested as a central mechanism. Here, we assessed COVID-19 post-infectious symptoms (5–6 months after PCR-confirmed acute infection) together with the humoral immune response against SARS-CoV-2 in non-hospitalized COVID-19 convalescents, early (5–6 weeks) and late (5–6 months) after their first positive SARS-CoV-2 PCR result. Convalescents reporting several post-infectious symptoms (>3) showed higher anti-spike and anti-nucleocapsid antibody levels 5–6 weeks after PCR-confirmed infection with the latter remained increased 5–6 months after positive PCR. Likewise, a higher post-infectious symptom score was associated with increased antibody levels. Of note, convalescents displaying neuro-psychiatric symptoms such as restlessness, palpitations, irritability, and headache, as well as general symptoms such as fatigue/reduced power had higher SARS-CoV-2-specific antibody levels compared with asymptomatic cases. The increased humoral immune response in convalescents with post-COVID syndrome might be useful for the detection of individuals with an increased risk for post-COVID syndrome.

## 1. Introduction

Severe acute respiratory syndrome coronavirus 2 (SARS-CoV-2), causing coronavirus disease 2019 (COVID-19), is still spreading around the world, so far reaching more than 600 million documented cases [1]. With approval of effective SARS-CoV-2 vaccines, the number of life-threatening COVID-19 courses has largely been reduced. However, multiple COVID-19 convalescents, even after asymptomatic to moderate disease, suffer from post-COVID syndrome with relevant limitations in daily life [2]. Recently, it was reported that after a mild course of COVID-19 11% of patients still could not fully participate in everyday and work life seven months after disease onset [3]. One of the key challenges for health systems in general and for physicians, dealing with post-COVID patients in particular, is the identification of COVID-19 patients who are at high risk for developing post-COVID syndrome [4].

Post-COVID syndrome is defined as the occurrence or continuation of post-infectious symptoms 3 months after COVID-19 that are not explained by an alternative cause [5,6,7]. The clinical course of post-COVID syndrome is very variable, with frequently reported symptoms being fatigue, dyspnea, anosmia, ageusia, headache, muscle pain, arthralgia, cough, cognitive impairment, and insomnia [8,9,10,11,12,13,14,15,16]. Patients experiencing severe COVID-19 are at increased risk for post-COVID syndrome, whereas the risk is lower for convalescents after asymptomatic to moderate COVID-19 [13,14,15,16]. A large population survey reported the prevalence of post-COVID syndrome in approximately 73% of hospitalized and 46% of non-hospitalized convalescents over 12 weeks after acute COVID-19 infection [14]. Thus far, the pathophysiological mechanisms of post-COVID syndrome are not fully understood. Direct viral toxicity, endothelial damage, endocrine alterations, dysregulation of the renin–angiotensin–aldosterone system, and dysregulated immune responses, including the development of autoantibodies, have been discussed as probable mechanisms [6,17,18,19,20,21,22,23,24,25]. While the development of the SARS-CoV-2-directed humoral immune response, in terms of the induction of SARS-CoV-2-specfic antibodies, is required for viral clearance in infected individuals and has been reported to be increased in patients with severe COVID-19 [26,27,28,29,30,31] the association of humoral immune response and post-COVID syndrome, in particular in non-hospitalized COVID-19 convalescents, is still sparse and even contradictory [3,32,33,34,35,36,37,38]. In this study, we analyzed the occurrence of different post-infectious symptoms (5–6 months after the first positive PCR result) related to early (5–6 weeks after PCR-confirmed COVID-19) and late (5–6 months after positive PCR) humoral immune response against SARS-CoV-2 in 51 non-hospitalized COVID-19 convalescents.

## 2. Materials and Methods

### 2.1. Participants and Serum Sample Collection

Serum samples from convalescent adults after asymptomatic, mild or moderate SARS-CoV-2 infection according to WHO criteria [39] (*n* = 51) were collected at the University Hospital Tübingen between April 2020 and August 2020. Informed consent was obtained in accordance with the Declaration of Helsinki protocol. The study was approved by and performed according to the guidelines of the local ethics committees (179/2020/BO2). SARS-CoV-2 infection was confirmed by polymerase chain reaction (PCR) test after nasopharyngeal swab. Serum samples were taken 35–56 days (T_E_ “early” timepoint) and 141–183 days (T_L_ “late” timepoint) after positive PCR (Table 1). Data on antibody levels and post-infectious symptoms were retrieved from previous publications [28,40].

### 2.2. Assessment of Post-Infectious Symptoms

A questionnaire-based assessment of ten post-infectious symptoms, including grading of severity (no, mild, moderate and severe), was performed at T_L_. Symptoms were classified into four categories: general symptoms (“fatigue” and “reduced performance”), altered sensory perception (“anosmia/ ageusia” and “hearing loss”), neuro-psychiatric symptoms (“restlessness”, “palpitations”, “irritability”, and “headache”), and pulmonary symptoms (“cough” and “dyspnea”). Patients were included in a category if at least one symptom for that category was reported by the patient. The symptom score was calculated by summing up the self-reported grading (no = 0, mild = 1, moderate = 2, severe = 3) of all of the reported symptoms. 

### 2.3. Antibody Responses

Anti-SARS-CoV-2 nucleocapsid antibody and anti-SARS-CoV-2-spike IgG-antibody titers were determined at T_E_ and T_L_. Data on anti-SARS-CoV-2 nucleocapsid antibody index values (including IgG and IgA) were assessed by Elecsys^®^ anti-SARS-CoV-2 immunoassay (Roche Diagnostics, Basel, Switzerland) and anti-SARS-CoV-2-spike IgG-antibody titers were assessed by Euroline Anti-SARS-CoV-2^®^ (Euroimmune, Luebeck, Germany).

The 96-well Euroline Anti-SARS-CoV-2^®^ (Euroimmune, 2606A_A_DE_C03, as constituted on 22 April 2020) was performed on an automated BEP 2000 Advance system (Siemens Healthcare Diagnostics GmbH) according to the manufacturer’s instructions. The ELISA assay detected anti-SARS-CoV-2 IgG directed against the S1 domain of the viral spike protein (including the immunologically relevant RBD) and relied on an assay-specific calibrator to report a ratio of specimen absorbance to calibrator absorbance. The final interpretation of positivity was determined by a ratio above a threshold value given by the manufacturer: positive (ratio ≥ 1.1), borderline (ratio 0.8–1.0), or negative (ratio < 0.8). The levels of anti-spike IgG-antibodies are shown in the graphs as a ratio above the threshold value. Quality control was performed following the manufacturer’s instructions on each day of testing.

The Elecsys^®^ anti-SARS-CoV-2 immunoassay is an ECLIA (electro-generated chemiluminescence immunoassay) designed by Roche Diagnostics GmbH and was used according to the manufacturer’s instructions (V1.0, as constituted in May 2020). It is intended for the detection of high-affinity antibodies (including IgG and IgA) directed against the nucleocapsid protein of SARS-CoV-2 in human serum. Readout was performed on the Cobas e411 analyzer (Roche Diagnostics). Negative results were defined by a cut-off index of <1.0. The levels of anti-nucleocapsid antibodies are shown in the graphs as an index value. Quality control was performed following the manufacturer’s instructions on each day of testing. 

Data on antibody levels and post-infectious symptoms were retrieved from previous publications [28,40].

### 2.4. Software and Statistical Analysis

Data are displayed as mean with standard deviation (SD), box plots with median and 25th and 75th quartile, min/max whiskers, and individual data points. Groups were tested using the Mann–Whitney U test or Kruskal–Wallis test and were corrected for multiple comparison if applicable. Statistical analyses were conducted using JMP Pro (SAS Institute, v.16, Cary, NC, USA) software. *p*-values < 0.05 were considered statistically significant. Missing data were denoted in tables and in the descriptive analysis. Graphs were plotted using GraphPad Prism v.9.1.2 (San Diego, CA, USA).

## 3. Results

### 3.1. Clinical Characteristics of COVID-19 Convalescents and Prevalence of Post-Infectious Symptoms

For this study, we analyzed post-infectious symptoms after SARS-CoV-2 infection at T_L_ (5–6 months after PCR-confirmed COVID-19) with SARS-CoV-2-specific antibody responses at T_E_ (5–6 weeks after positive PCR) and T_L_ of 51 convalescent individuals with an asymptomatic to moderate COVID-19 course. The mean age of convalescents at the time of infection was 43.4 (SD ± 13.7) years, with an equal gender distribution (female to male ratio of 1.04:1). No convalescent in this study had severe symptoms according to WHO criteria and no one was hospitalized due to COVID-19 infection [39]. Most convalescents (76%) reported at least one post-infectious symptom at T_L_ (median 2, range 0 to 9 symptoms, Table 1).

Here, 41%, 35%, and 24% of convalescents reported ≥ three, one to two, or no post-infectious symptoms, respectively (Table 1). The most frequent post-infectious symptoms were “fatigue” (45%), “anosmia and ageusia” (29%), “headache” (28%), “reduced performance” (28%), and “irritability” (21%). Pulmonary post-infectious symptoms such as “dyspnea” and “cough” were reported by 16% and 12% of convalescents, respectively (Table 1). A combinatorial symptom score of the number of different symptoms and their severity was determined. This score of > three, one to two, and 0 points was observed in 37%, 39%, and 24% of convalescents, respectively (range 0–17 points, median 3) (Table 1). Interestingly, only 14 of the convalescents attributed their symptoms as being post-infectious. For further analysis, all reported (post-infectious) symptoms were evaluated. In this work, convalescents with ≥ three symptoms (41%) or a symptom score > three points (37%) were considered as presenting with post-COVID syndrome (Table 1). Further details on convalescents’ characteristics are presented in Table 1.

### 3.2. Anti-SARS-CoV-2 Antibody Levels and Number of Post-Infectious Symptoms

Anti-SARS-CoV-2 anti-nucleocapsid and anti-spike antibody levels were assessed at T_E_ and T_L._ 84% and 75% of convalescents were positive for anti-spike antibodies at T_E_ and T_L_, respectively, whereas positivity for anti-nucleocapsid antibodies remained stable with a seroconversion rate of 88% at both timepoints (Table 1).

**Figure 1:**
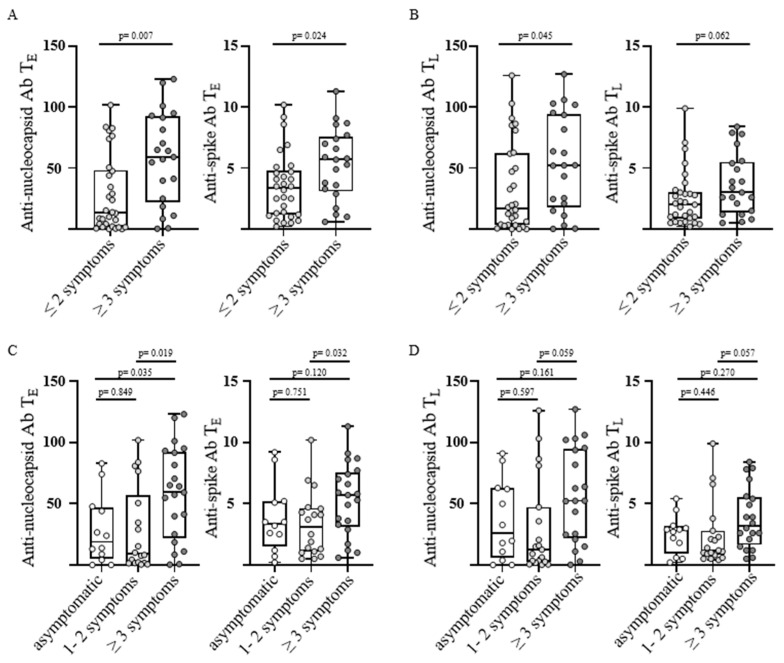
Number of post-infectious symptoms and antibody response in SARS-CoV-2 convalescents. Anti-nucleocapsid antibody (Ab) (left) and anti-spike Ab levels (right) were assessed in convalescent donors (n = 51) at T_E_ (“early” timepoint 5–6 weeks after positive PCR, (**A**,**C**)) and T_L_ (“late” timepoint 5–6 months positive PCR, (**B**,**D**)). Convalescents were grouped according to the number of post-infectious symptoms at T_L_. Levels of anti-spike Ab are shown as a ratio above the threshold value. Levels of anti-nucleocapsid Ab are shown as an index value. Data are presented as box plots with 25th and 75th percentiles and min/max whiskers. *p*-values were calculated using the Mann–Whitney U test (**A**,**B**) and Kruskal–Wallis test (**C**,**D**). *p*, *p*-value; Ab, antibody.

To assess SARS-CoV-2-directed humoral immune responses in convalescents with different post-infectious symptoms, convalescents were grouped according to the number of reported symptoms into two groups (≤2 symptoms (n = 30) versus ≥3 symptoms (n = 21) corresponding to post-COVID syndrome). Convalescents with post-COVID syndrome displayed significantly increased anti-nucleocapsid antibody levels at T_E_ and T_L_, as well as significantly increased anti-spike-antibody levels at T_E_, as well as a trend for increased anti-spike levels at T_L_ (*p*-values: 0.007, 0.045, 0.024, and 0.062, respectively; Figure 1A,B).

In the next step, we divided the group of convalescents without post-COVID-syndrome into asymptomatic individuals and individuals reporting one to two symptoms to further investigate the differences in SARS-CoV-2-specific humoral immune response especially between these two populations. Therefore, three groups were evaluated (asymptomatic, 1–2 symptoms and ≥3 symptoms corresponding to post-COVID syndrome). In line with the previous findings, convalescents reporting post-COVID syndrome had significantly increased anti-nucleocapsid antibody levels at T_E_ in comparison with both asymptomatic individuals and convalescents with only one to two symptoms (*p*-values: 0.035 and 0.019, respectively; Figure 1C), with a similar trend at T_L_ (*p*-values: 0.161 and 0.059, respectively; Figure 1D). Anti-spike antibody levels were similarly increased in convalescents reporting many symptoms (≥3) at T_E_, being only significant in between strongly affected and mildly affect individuals (*p*-value: 0.032; Figure 1C). At T_L_ the same trend for higher anti-spike antibody levels in individuals with post-COVID syndrome was observed, failing to reach significance in comparison to asymptomatic or mildly affected convalescents (*p*-values: 0.270 and 0.057, respectively; Figure 1D). Of note, there were no significant differences between asymptomatic individuals and convalescents with one to two symptoms for anti-nucleocapsid and anti-spike antibody levels at both timepoints T_E_ and T_L_ (*p*-values: 0.849, 0.597, 0.751, and 0.446; Figure 1C,D).

Taken together, our data showed elevated SARS-CoV-2-specific humoral immune response in individuals with post-COVID syndrome (≥3 symptoms) in comparison with both asymptomatic and mildly affected convalescents, whereas the antibody levels did not differ significantly between the last two mentioned.

### 3.3. Anti-SARS-CoV-2 Antibody Levels and Severity of Post-Infectious Symptoms

**Figure 2:**
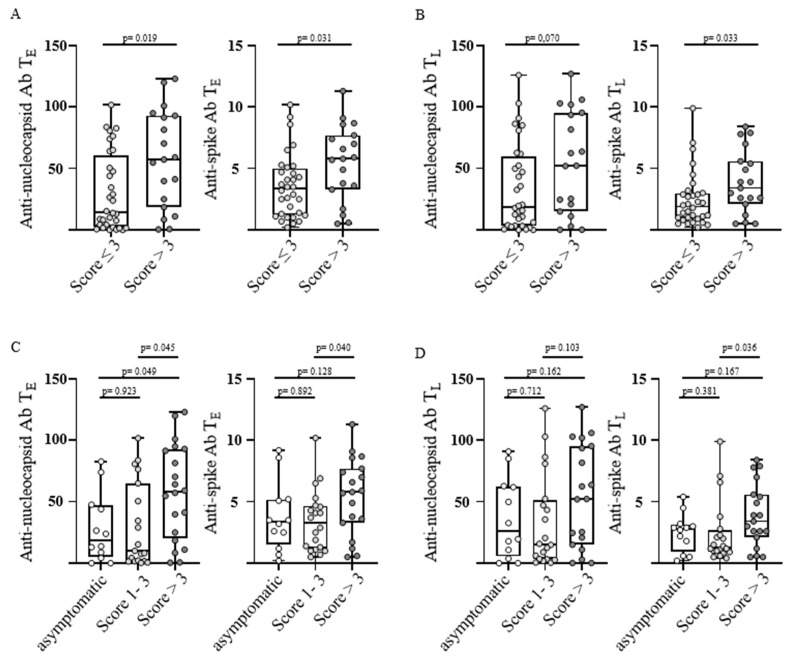
Severity of post-infectious symptoms and antibody response after SARS-CoV-2 infection. Anti-nucleocapsid antibody (Ab) levels (left) and anti-spike Ab levels (right) were assessed in COVID-19 convalescent donors (n = 51) at T_E_ (“early” timepoint 5–6 weeks after positive PCR, (**A**,**C**)) and T_L_ (“late” timepoint 5–6 months after positive PCR, (**B**,**D**)). Convalescents were grouped according to post-infectious symptom severity at T_L_, as defined by the symptom score. The existence and severity (no, mild, moderate, and severe) of 10 individual symptoms were assessed using a questionnaire. The symptom score was determined by adding up the gradings of all single symptoms according to self-reported grading (no = 0, mild = 1, moderate = 2, severe = 3). The levels of anti-spike Ab are shown as a ratio above the threshold value. Levels of anti-nucleocapsid Ab are shown as an index value. Data are presented as box plots showing the median with 25th and 75th percentiles and min/max whiskers. *p*-values were calculated using the Mann–Whitney U test (**A**,**B**) and Kruskal–Wallis test (**C**,**D**). *p*, *p*-value; Ab, antibody.

To assess humoral immune response and the severity of post-infectious symptoms convalescents were grouped according to the symptom score into two groups (score ≤ 3 (n = 32) versus score > 3 (n = 19) corresponding to post-COVID syndrome). Convalescents with post-COVID-syndrome showed significantly higher anti-nucleocapsid antibody levels at T_E_ as well as significantly increased anti-spike antibody levels at T_E_ and T_L_ (*p*-values: 0.019, 0.031 and 0.033, respectively; Figure 2A,B).

To further investigate differences in SARS-CoV-2-specific humoral immune response, especially between asymptomatic individuals and individuals with a score of one to three, we divided the group of convalescents without post-COVID-syndrome into these two populations. Therefore, three groups were evaluated (asymptomatic with a score of 0, low with a score of 1–3, and high with a score >3 corresponding to post-COVID syndrome). As observed before, convalescents with a high symptom score had significantly increased anti-nucleocapsid antibody levels at T_E_ in comparison with both asymptomatic individuals and convalescents with a low symptom score (*p*-values: 0.049 and 0.045, respectively; Figure 2C), with a similar trend also at T_L_ (*p*-values: 0.162 and 0.103; Figure 2D). Anti-spike antibody levels were increased likewise in convalescents with a high symptom score in comparison to both other groups at T_E_ and T_L_, being significant only when compared with mildly affected convalescents at both time points (*p*-values: 0.128, 0.040, 0.167, and 0.036; Figure 2C,D). Of note, there were no significant differences between asymptomatic individuals and convalescents with a low symptom score for anti-nucleocapsid or anti-spike antibody titers at any time point (*p*-values: 0.923, 0.712, 0.892, and 0.381; Figure 2C,D).

In summary, we observed increased SARS-CoV-2-specific antibody levels in individuals with post-COVID syndrome (symptom score > 3) in comparison to both asymptomatic and mildly affected convalescents, whereas no differences could be found in between the last two mentioned.

### 3.4. Anti-SARS-CoV-2 Antibody Levels and Single Post-Infectious Symptoms

**Figure 3:**
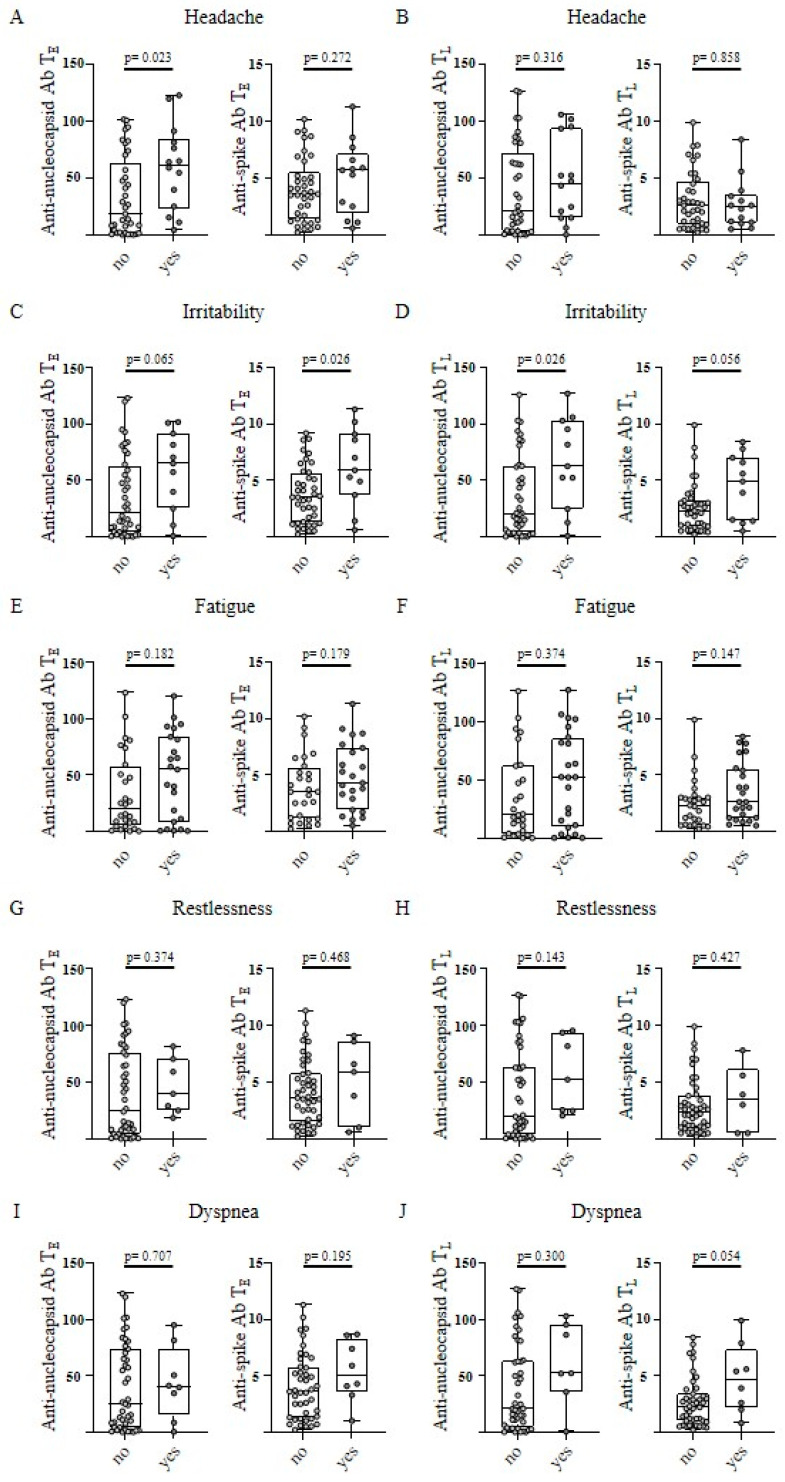
Single post-infectious symptoms in correlation with antibody response after SARS-CoV-2 infection. Anti-nucleocapsid antibody (Ab) levels (left) and anti-spike Ab levels (right) were assessed in COVID-19 convalescent donors (n = 51) at T_E_ (“early” timepoint 5–6 weeks after positive PCR, (**A**,**C**,**E**,**G**,**I**)) and TL (“late” timepoint 5–6 months after positive PCR, (**B**,**D**,**F**,**H**,**J**)). Convalescents were grouped according to the existence (no/yes) of single post-infectious symptoms at T_L_. The levels of anti-spike Ab are shown as a ratio above the threshold value. Levels of anti-nucleocapsid Ab are shown as an index value. Data are presented as box plots showing the median with 25th and 75th percentiles and min/max whiskers. *p*-values were calculated by Mann–Whitney U test. *p*, *p*-value; Ab, antibody.

In the next step, a potential association of SARS-CoV-2-directed humoral immune response with specific single post-infectious symptoms was assessed. Significant differences were observed for the single post-infectious symptoms “headache” and “irritability”. Convalescents reporting “headache” exhibited higher anti-nucleocapsid antibody levels at T_E_, with a similar trend at T_L_ (*p*-values: 0.023 and 0.316, respectively; Figure 3A). No relevant differences were shown for anti-spike antibody levels at T_E_ and T_L_ (*p*-values: 0.272 and 0.858, respectively; Figure 3B).

In terms of the symptom “irritability”, convalescents had significantly higher anti-nucleocapsid antibody levels at T_L_ and trended to elevated anti-nucleocapsid antibody levels at T_E_ (*p*-values; 0.026 and 0.065, respectively; Figure 3C). In addition, anti-spike antibody levels were significantly increased at T_E_ with a similar, but not significant, trend at T_L_ (*p*-values; 0.026 and 0.056; Figure 3D). For all other symptoms no relevant differences in anti-SARS-CoV-2 antibody levels were observed (Figure 3E–J, Figure S1A–H). 

### 3.5. Anti-SARS-CoV-2 Antibody Levels and Classification of Symptoms

**Figure 4:**
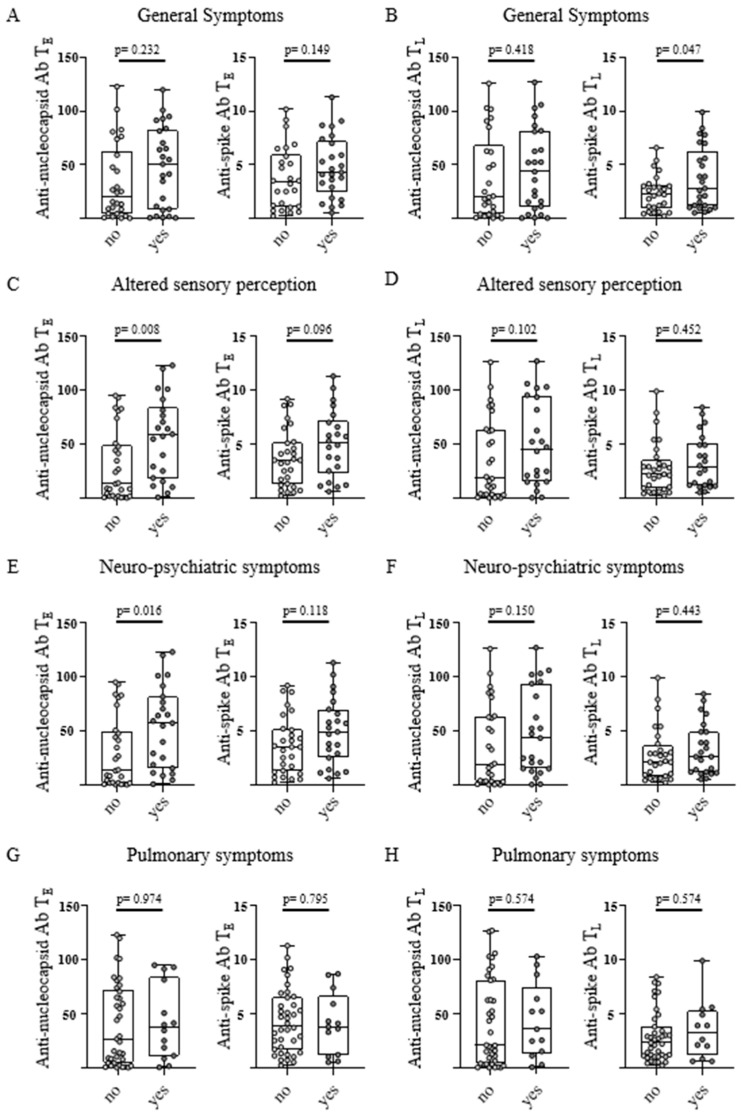
Classification of post-infectious symptoms in correlation with antibody response in SARS-CoV-2 convalescents. Anti-nucleocapsid antibody (Ab) levels (left) and anti-spike Ab levels (right) were assessed in COVID-19 convalescent donors (n = 51) at T_E_ (“early” timepoint 5–6 weeks after positive PCR, (**A**,**C**,**E**,**G**)) and T_L_ (“late” timepoint 5–6 months after positive PCR, (**B**,**D**,**F**,**H**) grouped according to the existence (no/yes) of categorized post-infectious symptoms at T_L_. The existence of single symptoms was assessed using a questionnaire. The categorization of single symptoms was based on the similar nature of the symptoms: general symptoms included fatigue and reduced performance; altered sensory perception combined anosmia, ageusia, and hearing loss; neuropsychiatric symptoms comprised restlessness, palpitations, irritability, and headache; and pulmonary symptoms included cough and dyspnea. Levels of anti-spike Ab are shown as a ratio above the threshold value. Levels of anti-nucleocapsid Ab are shown as an index value. Data are presented as box plots showing the median with 25th and 75th percentiles and min/max whiskers showing all points. *p*-values were calculated using the Kruskal–Wallis-test. *p*, *p*-value; Ab, antibody.

For further analysis, post-infectious symptoms were classified according to general symptoms, altered sensory perception, neuro-psychiatric, and pulmonary symptoms. The appearance of general symptoms (“fatigue” and “reduced performance”) was observed in convalescents with significantly higher anti-spike antibody levels at T_L_, with the same trend at T_E_ (*p*-values: 0.047 and 0.149, respectively; Figure 4A,B).

Individuals that reported altered sensory perceptions (“anosmia/ageusia” and “hearing loss”) had significantly higher anti-nucleocapsid antibody levels at T_E_, with the same tendency at T_L_ (*p*-values: 0.008 and 0.102, respectively; Figure 4C,D). Convalescents with a neuro-psychiatric symptom (“restlessness”, “palpitations”, “irritability”, and “headache”) showed significantly higher anti-nucleocapsid antibody levels at T_E_ with a similar trend at T_L_ (*p*-values: 0.016 and 0.150, respectively; Figure 4E,F). A tendency towards higher anti-spike antibody levels could also be noticed at T_E_ in this classification (*p*-values: 0.118; Figure 4E). Interestingly, for pulmonary symptoms (“cough” and “dyspnea”) no significant differences in anti-nucleocapsid and anti-spike antibody levels were observed at both time points (*p*-values: 0.974, 0.574, 0.795 and 0.574, respectively; Figure 4G,H).

## 4. Discussion

The clinical course of post-COVID syndrome can be very variable and it is frequently reported even in non-hospitalized convalescents with an asymptomatic to moderate course of COVID-19 [14,15,16]. In this work, we observed post-COVID syndrome in about 40% of all individuals 5–6 months after PCR-confirmed acute infection, as well as increased anti-SARS-CoV-2-specific antibody levels (5–6 weeks and 5–6 months after positive PCR) in post-COVID syndrome patients. In addition, convalescents reporting general symptoms, altered sensory perceptions, or neuropsychiatric symptoms showed higher anti-SARS-CoV-2-specific antibody levels.

In this non-hospitalized group of convalescents, 76% of individuals reported at least one post-infectious symptom 5–6 months after PCR-confirmed COVID-19, which was higher compared with another study reporting at least one post-infectious symptom in about 40% of convalescents six months after positive PCR [33]. Of note, in hospitalized convalescents similar rates (73%) of post infectious symptoms were observed [14]. Differences in the prevalence of post-COVID syndrome were most likely due to differing assessment with regards to time points and methods, divergent definitions of post-COVID syndrome, and the baseline characteristics of the convalescents [9,10,14,33,37,41]. Currently, the definition of post-COVID syndrome relies on the persistence of symptoms, which cannot be explained by an alternative cause 3 months after COVID-19 and a general definition of symptoms for the diagnosis is missing [42]. In this study, most convalescents reporting symptoms 5–6 months after PCR confirmed acute infection attributed symptoms to be post-infectious after COVID-19. Of note, this study was conducted early in the SARS-CoV-2 pandemic and was influenced by a lack of awareness of post-COVID syndrome and in addition, the self-reported assessment of symptoms by questionnaire has to be taken with caution [43,44]. Therefore, all of the reported (post-infectious) symptoms were evaluated and convalescents with more than two symptoms or a symptom score > 3 were considered as presenting with post-COVID syndrome. This rate is in line with a prospective and longitudinal study reporting that 35% of non-hospitalized COVID-19 patients suffer from post-COVID syndrome 7 months after infection [3]. The most frequently documented symptoms in our cohort were “fatigue”, “anosmia and ageusia”, “headache”, and “reduced performance”, as previously reported [14].

Post-COVID syndrome significantly limits the activity of daily life and can thus lead to tremendous socioeconomical consequences [2,4]. Therefore, a key challenge for physicians is the identification of COVID-19 patients being at high risk of developing post-COVID syndrome [4]. So far, the cause of post-COVID syndrome is not fully understood and is even discussed contradictory, which further complicates the search for reliable predictive markers [6,17,18,19,20,21,22,23,24,25]. Some risk factors for post-COVID syndrome have been identified, such as age, body mass index, gender, and severity of symptoms during acute COVID-19 [45,46,47,48]. In addition, our and other studies have reported on differential SARS-CoV-2-specific humoral immune responses in convalescents with post-COVID syndrome and the role of the SARS-CoV-2-specific humoral immune response in post-COVID syndrome is still a matter of debate as data are incomplete and even partially contradictory, which may be attributable to the different methodological approaches [3,32,33,34,35,36,37,38]. In our cohort, 84% and 75% of convalescents were positive for anti-spike antibodies at T_E_ and T_L_, respectively, whereas positivity for anti-nucleocapsid antibodies remained stable with a seroconversion rate of 88% at both timepoints. In this work, we used the Elecsys^®^ anti-SARS-CoV-2 anti-N immunoassay (Roche Diagnostics) for the detection of anti-nucleocapsid antibodies. This assay is known to be more sensitive during the complete course of a SARS-CoV-2 infection compared to many other assays, which is consistent with our results [49,50]. In addition, the competitive Elecsys^®^ immunoassay showed longer seropositivity rates, while antibody titers over time decreased over time [40,49,50]. This is most likely explained by the influence of antibody avidity and the relative abundance of the different immunoglobulin classes in the competitive assay [49,50,51]. Recently, increased SARS-CoV-2-specific antibody levels (anti-spike and anti-nucleocapsid) were observed in patients with post-COVID syndrome 6 months after infection [33]. Likewise, we could show that anti-nucleocapsid antibody levels 5–6 months after PCR-confirmed acute infection were associated with post-COVID syndrome, significantly for the quantity and tendentially for the severity of the experienced post-infectious symptoms. In addition, we observed significantly increased anti-nucleocapsid antibody levels in post-COVID syndrome patients at an earlier time point 5–6 weeks after positive PCR, which might help to identify convalescents with an increased risk for post-COVID syndrome, as anti-nucleocapsid antibodies are not induced by most of the EMA-approved vaccines. Moreover, faster symptom resolution of acute COVID-19 is associated with higher anti-nucleocapsid antibody levels during the first week after diagnosis, which additionally underlines their importance [37]. 

In this work, we observed significantly increased anti-spike-antibody levels early and late after acute infection in convalescents with post-COVID syndrome, while the overall anti-spike-antibodies in all convalescents decreased over time. This is consistent with reports by others showing that anti-spike antibody levels wane over time and convalescents with more and more severe post-infectious symptoms have higher anti-spike antibody levels after infection [26,27,28,29,30,31,32]. Our results resemble the observations made by Durstenfeld et al. reporting on higher anti-receptor-binding-domain antibody levels in convalescents with post-infectious cardio-pulmonary symptoms 7 months after acute infection [35]. 

Besides the number and severity of post-infectious symptoms, we also assessed the character of post-infectious symptoms with SARS-CoV-2-specific humoral immune response. Convalescents reporting post-infectious neuropsychiatric symptoms had significantly higher anti-nucleocapsid antibody levels 5–6 weeks after PCR-confirmed COVID-19. This is of special importance, as post-infectious neuropsychiatric symptoms have been observed in many convalescents suffering from post-COVID syndrome [41,52]. In addition, we showed in convalescents with altered sensory perception significantly higher anti-nucleocapsid antibody levels, including both IgG and IgA, 5–6 weeks after positive PCR. Interestingly, others reported on lower anti-spike IgA antibody levels in convalescents with persisting post-infectious up to 3 months after acute infection anosmia or ageusia, highlighting the importance of IgA antibodies on mucosal tissue [38].

With increasing reports of post-COVID syndrome and post-infectious fatigue in SARS-CoV-2 convalescents, COVID-19, similar to many other viral infections (e.g., Epstein–Barr virus, human herpes virus-6, or the human parvovirus B19), has become increasingly discussed as a trigger for Myalgic Encephalomyelitis/Chronic Fatigue Syndrome (ME/CFS) [53,54]. In this study, we observed higher anti-spike antibody levels early after COVID-19 infection in convalescents reporting post-infectious “fatigue” and/or a “reduced performance”. Of note, a post-infectious dysregulated immune response and autoimmunity, including the development of autoantibodies, have been intensively discussed as a key feature of the ME/CFS pathophysiology [53,55,56].

Together, this study provides further insights into the association of SARS-CoV-2-specific humoral immune response with post-COVID syndrome: increased SARS-CoV-2-specific antibody levels were observed early after infection in convalescents with more and more severe post-infectious symptoms approximately 6 months after infection, in particular in individuals suffering from neuro-psychiatric symptoms. Based on this observation, it is tempting to hypothesize that assessment of the humoral immune response, including anti-nucleocapsid antibody levels, can help to identify convalescents who are at increased risk for post-COVID syndrome. 

## Figures and Tables

**Table 1:** Donor characteristics and post-infectious symptoms of COVID-19 convalescent donors.

Donors	
Number	51
**Age (years)**	
Mean	43.3
SD	13.7
**Sex (n [%])**	
Female	26 (51)
Male	25 (49)
**Number of post-infectious symptoms at T_L_ (n)**	
Median	2
Range	0–9
**Number of post-infectious symptoms at T_L_ (n [%])**	
≥ 3	21 (41)
1–2	18 (35)
0	12 (24)
**Symptom score at T_L_ (score value)**	
Median	3
Range	0–17
**Distribution of symptom score at T_L_ (n [%])**	
>3	19 (37)
1–3	20 (39)
0	12 (24)
**Self-classification of donors at T_L_ (n [%])**	
Asymptomatic donors	12 (24)
Symptomatic donors	39 (76)
- attributed to COVID-19	14 (27)
**Single symptoms at T_L_ (n [%])**	
Fatigue	23 (45)
Anosmia and ageusia	15 (29)
Headache	14 (28)
Reduced performance	14 (28)
Irritability	11 (22)
Dyspnea	8 (16)
Restlessness	7 (14)
Palpitations	7 (14)
Cough	6 (12)
Hearing loss	5 (10)
**SARS-CoV-2 antibody test positivity (n [%])**	
Anti-spike antibody T_E_	43 (84)
Anti-spike antibody T_L_	38 (75)
Anti-nucleocapsid antibody T_E_	45 (88)
Anti-nucleocapsid antibody T_L_	45 (88)

n: number. SD: standard deviation. T_E_: “early” timepoint 5–6 weeks after positive PCR. T_L_: “late” timepoint 5–6 month after positive PCR.

## Data Availability

The data presented in this study are available upon request from the corresponding author.

## Supplementary Materials

The following supporting information can be downloaded at: https://www.mdpi.com/article/10.3390/v15030701/s1, Figure S1: Additional single post-infectious symptoms in correlation to antibody response after SARS-CoV-2 Infection.
