# Supplementary material for: Elevated SARS-CoV-2-Specific Antibody Levels in Patients with Post-COVID Syndrome
Source: Viruses. 2023 Mar 8;15(3):701. doi: 10.3390/v15030701 (PMC10051370; doi:10.3390/v15030701)
Supplement: Supplementary file 1 [file viruses-15-00701-s001.zip › viruses-2247552-supplementary.pdf]

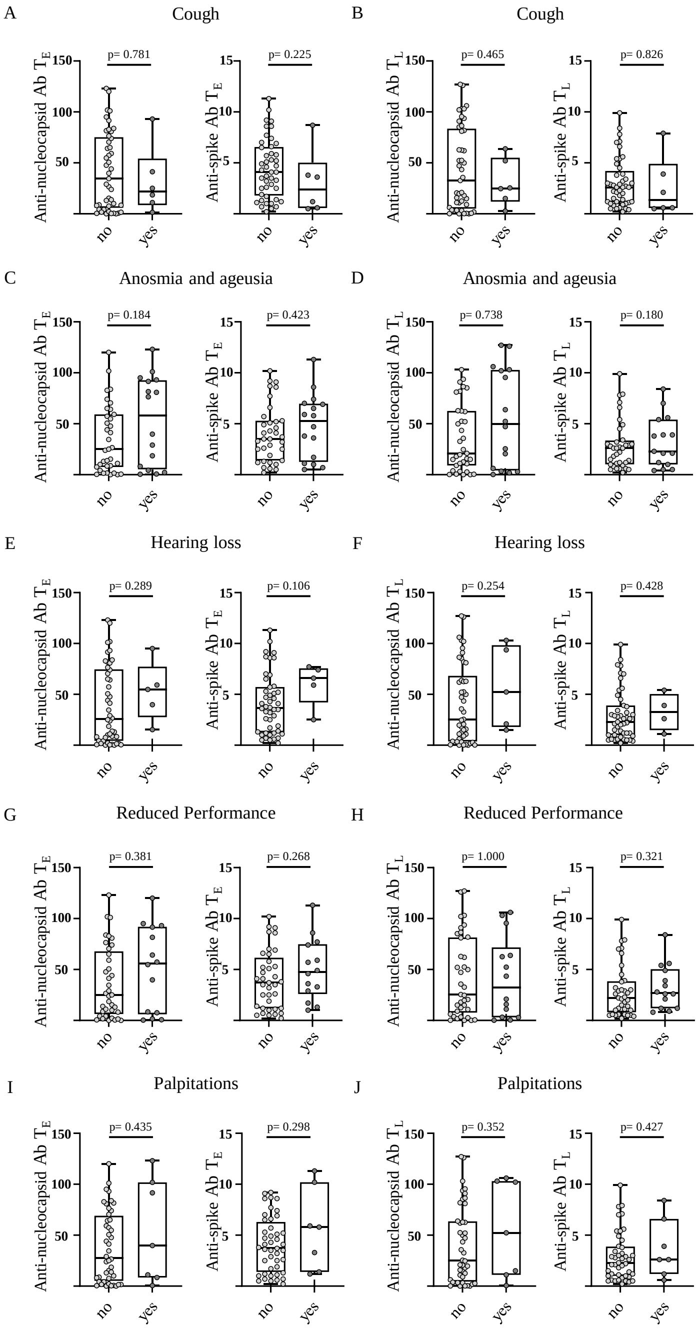

Supplemental Figure S1. Additional single post-infectious symptoms in correlation to antibody response after SARS-CoV-2 Infection. Anti-nucleocapsid antibody (left) (Ab) levels and anti-spike Ab levels (right) were assessed in COVID-19 convalescent donors (n= 51) at TE ("early" timepoint 5-6 weeks after infection, A, C, E, G and I) and TL ("late" timepoint 5-6 months after infection, B, D, F, H and J). Convalescents were grouped according to existence (no/yes) of single post-infectious symptoms at TL. Data are presented as box plots showing the median with 25th and 75th percentiles and min/max whiskers. P-values were calculated by Mann-Whitney-U test. p, p-value; Ab, antibody.
